# Potential Anti-*Candida albicans* Mechanism of Trichoderma Acid from *Trichoderma spirale*

**DOI:** 10.3390/ijms24065445

**Published:** 2023-03-13

**Authors:** Wei Ye, Yuchan Chen, Weimin Zhang, Taomei Liu, Yuping Liu, Mengran Li, Saini Li, Liqiong Xu, Hongxin Liu

**Affiliations:** State Key Laboratory of Applied Microbiology Southern China, Guangdong Provincial Key Laboratory of Microbial Culture Collection and Application, Institute of Microbiology, Guangdong Academy of Sciences, No. 100 Xianlie Middle Road, Yuexiu District, Guangzhou 510070, China

**Keywords:** *Candida albicans*, trichoderma acid, transcriptomic, proteomic, mitochondrial

## Abstract

*Candida albicans* is the main causal pathogen of fungal infections in human beings. Although diverse anti-*C. albicans* drugs have been explored, the drug resistance and side effects of these drugs are intensifying. Thus, it is urgent to explore new anti-*C. albicans* compounds from natural products. In this study, we identified trichoderma acid (TA), a compound from *Trichoderma spirale* with a strong inhibitory effect on *C. albicans*. Transcriptomic and iTRAQ-based proteomic analyses of TA-treated *C. albicans* in combination with scanning electronic microscopy and reactive oxygen species (ROS) detection were performed to investigate the potential targets of TA. The most significant differentially expressed genes and proteins after TA treatment were verified through Western blot analysis. Our results revealed that mitochondrial membrane potential, endoplasmic reticulum, ribosomes in the mitochondria, and cell walls were disrupted in TA-treated *C. albicans*, leading to the accumulation of ROS. The impaired enzymatic activities of superoxide dismutase further contributed to the increase in ROS concentration. The high concentration of ROS led to DNA damage and cell skeleton destruction. The expression levels of Rho-related GTP-binding protein RhoE (RND3), asparagine synthetase (ASNS), glutathione S-transferase, and heat shock protein 70 were significantly up-regulated in response to apoptosis and toxin stimulation. These findings suggest that RND3, ASNS, and supereoxide dismutase 5 are the potential targets of TA, as further demonstrated through Western blot analysis. The combination of transcriptomic, proteomic, and cellular analyses would provide clues for the anti-*C. albicans* mechanism of TA and the defensive response mechanism of *C. albicans*. TA is thus recognized as a promising new anti-*C. albicans* leading compound that alleviates the hazard of *C. albicans* infection in human beings.

## 1. Introduction

*Candida albicans* is one of the main pathogenic fungi causing a wide spectrum of disease in human beings. It mainly colonizes the human mucous membrane. It can cause superficial or invasive infections. Moreover, in humans, it can cause deep tissue infection and even mortality [1]. In recent years, *C. albicans* infections have continually increased due to the abuse of antibiotics. The pathogenic mechanism of *C. albicans* has been widely investigated. The pathogenicity of *C. albicans* is closely associated with the morphological transformation of *C. albicans*. Genes in the mitogen-activated protein kinase (MAPK) pathway, including *CPH1*, *HST7*, *CST20*, and *CEK1*, have been reported to be responsible for the morphological changes and hyphal formation of *C. albicans.* Strains with the deletion of these genes have considerably shorter hyphae than wild-type strains [2,3,4]. Various kinds of enzymes, including hydrolases, lipases, and proteases, especially secretory acid protease, have been demonstrated to be important virulence factors for *C. albicans* [5].

Diverse natural products with anti-*C. albicans* activities have been identified [6,7]. Azole drugs have been clinically used to treat epidermal and deep infections caused by *C. albicans* due to their advantages of broad-spectrum action and good tolerance [8,9]. However, azole drugs also cause side effects, such as abnormal visual and liver functions. Thus, exploiting novel natural products as leading anticandidal drug compounds is urgent [10,11,12]. Various flavonoids show strong inhibitory effects on *C. albicans* by inhibiting the efflux pump, inducing apoptosis (baicalein and sedonan A), cell wall damage (catechins), and disrupting the cytoplasmic membrane (carvacrol) (7, 10). Retigerc acid B can promote the expression of the *Dpp3* gene in *C. albicans* and facilitate the secretion of farnesol [13]. Retigerc acid B shows potent anticandidal activity via the inhibition of the formation of *C. albicans* hyphae, the expression of adhesion- and invasion-related factors, the initiation of the burst of reactive oxygen species (ROS), and the induction of mitochondrial membrane potential hyperpolarization via the Ras1–cAMP–Efg1 signal regulation pathway [13]. Dictamnine inhibits the growth of *C. albicans* in vitro by repressing its biofilm activity [14]. Phospholipase B enhances the permeability of *C. albicans* by decomposing the phospholipids of the *C. albicans* cell membrane [8]. Candidalysin, the first toxin extracted from *C. albicans*, is produced when *C. albicans* forms invasive hyphae, thus facilitating cell membrane penetration and aggravating infection [15]. Tian et al. reported that nerol led to the release of cytochrome C [16], the activation of caspase protein, and the increase in intracellular calcium ion and ROS contents, thus rapidly reducing the mitochondrial membrane potential of *C. albicans*.

Nevertheless, the progress of the development of drugs for infectious fungal diseases remains slow due to the severe side effects induced by antifungal drugs in the human body [16,17]. In our previous study, we isolated a compound named trichoderma acid (TA) from *Trichoderma spirale* A17, an endophytic fungus of *Aquilaria sinensis* [18]. In this study, we discovered that TA exhibited very strong inhibitory activity against *C. albicans*, which is stronger than that of the positive control nystatin. Furthermore, TA showed very weak cytotoxicity against human normal cells LX-2, indicating the great potential of TA as an anticandidal leading compound. TA is composed of a hexahexane skeleton and a unique conjugated pentadienoic acid fat chain. This structure is obviously different from the structures of existing antifungal drugs, suggesting that TA has a unique inhibitory mechanism against *C. albicans*. In this study, transcriptome sequencing and proteome sequencing combined with flow cytometry assay, qRT-PCR, and Western blot analysis were employed to investigate the significantly differentially expressed genes and proteins between TA-treated and untreated *C. albicans*. This study could provide molecular clues for the development of TA as a new leading compound against *C. albicans.*

## 2. Results

### 2.1. Anti-C. albicans Activity of TA

**Figure 1:**
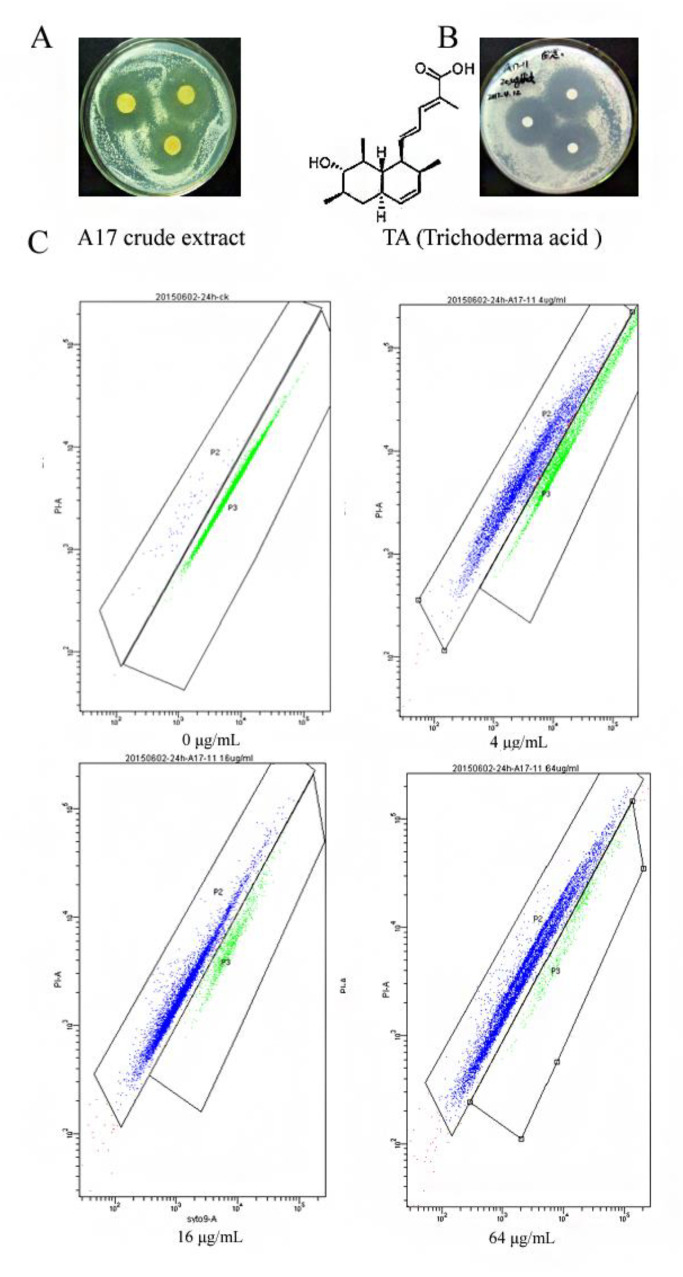
The anti-*C. albicans* effect of the extract from *Trichoderma spirale* and TA: (**A**) inhibitory effect of *Trichoderma spirale* A17 extract; (**B**) inhibitory effect of TA; (**C**) flow cytometry assay of the MIC of TA towards *C. albicans.* The results indicate the anti-candidal effect of the crude extract and TA. Additionally, the MIC_50_ value of TA was calculated by SigmaPlot.

The anti-candidal effect of crude extract from *T. spiralis* A17 and TA were evaluated. TA was isolated from the crude extract of the A17 strain. The chemical formula of TA is C_19_H_28_O_3_ with a molecular weight of 304.4140, and it is an octahydro-naphthalene derivative. TA has a skeleton containing two 6-6 conjugated rings and a unique long chain of conjugated glutaric acid fats (Figure 1) [18], and the structure of TA is the same as the compound (2E, 4E)-5-((1S, 2S, 4aR, 6R, 7S, 8S, 8aS)-7-hydroxy-2,6,8-trimethyl-1, 2, 4a, 5, 6, 7, 8a-octahydronaphthalen-1-yl)-2-methylpenta-2,4-dienoic acid isolated by Yamada et al. [19]. The crude extract of *T. spiralis* A17 showed strong anti-*C. albicans* activity with the concentration of 50 μg/mL (Figure 1A). The anticandidal activity of purified TA was also determined in this study. The diameter of the anti-*C. albicans* inhibition zone reached 23 mm under treatment with the concentration of 20 μg/mL TA (Figure 1B). The MIC values of TA against *C. albicans* were investigated through flow cytometry. The MIC_50_ and MIC_80_ of TA against *C. albicans* were 3.46 and 9.21 μg/mL, respectively (Figure 1C), which were stronger than those of positive control nystatin (5.18 μg/mL and 14.14 μg/mL, respectively), which is a clinical drug for the treatment of infections caused by *C. albicans*. The growth curves of *C. albicans* treated with TA indicated that TA significantly inhibited the growth of *C. albicans* (Figure S1), and the results indicated that the MIC_50_ of TA towards *C. albicans* is 4 μg/mL, which was in accordance with the flow cytometry results, implying the great potential of TA for development as a novel anti-*C. albicans* leading compound.

### 2.2. Cytotoxicity Assay of TA

TA was evaluated for its in vitro cytotoxicity against normal human-derived cell line LX-2 (human hepatic stellate cells). The results showed that the IC_50_ values of TA against LX-2 cell lines were 63.27 ± 1.31 μM, suggesting that TA did not exhibit obvious cytotoxicity towards human-derived cells.

### 2.3. Laser Confocal and Scanning Electron Microscopy and Observation of TA-Treated C. albicans

**Figure 2:**
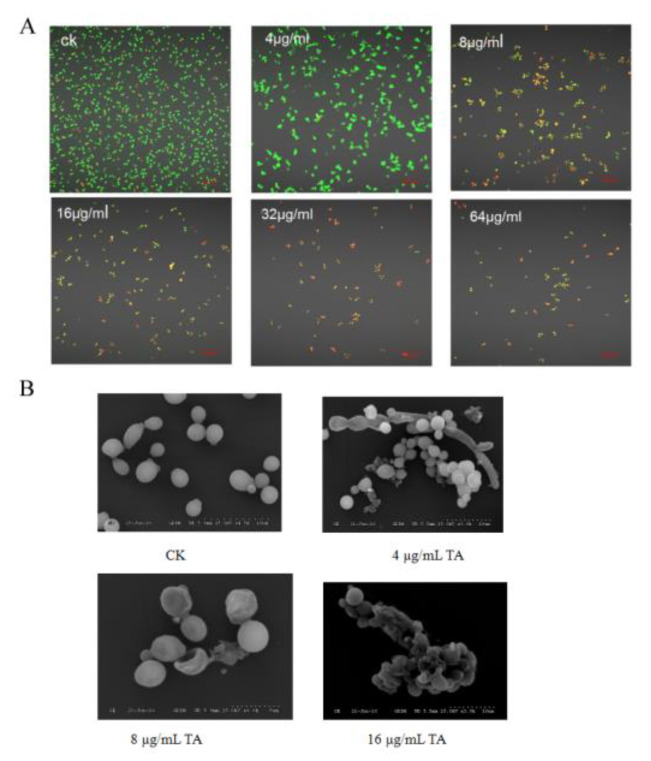
The observation of TA-treated *C. albicans* via laser confocal microscopy and scanning electronic microscopy: (**A**) Laser confocal observation. The magnification fold was ×400 fold with the scale of 50 μm; (**B**) SEM observation. The scale was 10 μm.

Laser confocal and scanning electron microscopy (SEM) were employed to observe the *C. albicans* cells after TA treatment. JC-1 dye was used to stain TA-treated *C. albicans*. The cells with a red color indicated dead cells. Laser confocal microscopy observation revealed that the number of living *C. albicans* cells decreased significantly after treatment with 4 μg/mL TA (TA-4). A large amount of *C. albicans* cells died after treatment with 8 μg/mL TA (TA-8), as indicated by the increase in red fluorescence intensity. Few living *C. albicans* cells were observed after treatment with 16 and 32 μg/mL TA (Figure 2A). The altered ratio of red fluorescence to green fluorescence after TA treatment indicated the destruction of the mitochondria and the decline of mitochondrial membrane potential.

Scanning electron microscopy (SEM) was utilized to observe TA-treated *C. albicans*. At 24 h after treatment, *C. albicans* cells treated with TA-4 began to shrink, approximately half of the *C. albicans* cells treated with TA-8 were disrupted, and nearly all 16 μg/mL TA-treated *C. albicans* cells were completely disrupted (Figure 2B). Nevertheless, the detailed mechanism underlying the effect of TA treatment on *C. albican* needs to be further investigated through the combination of transcriptomic sequencing and proteomic sequencing.

### 2.4. Detection of Mitochondrial Membrane Potential and ROS

**Figure 3:**
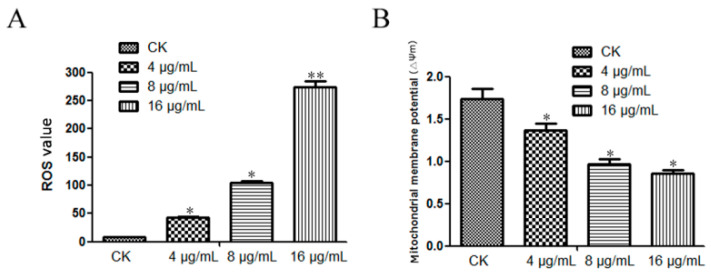
The ROS value and mitochondrial membrane potential detection: (**A**) ROS value; (**B**) mitochondrial membrane potential. The TA treatment can elevate the ROS value and down-regulate the mitochondrial membrane potential. “*” indicates *p* < 0.05, “**” indicates *p* < 0.01.

The ROS value and mitochondrial membrane potential were also detected to further analyze the anti-candidal mechanism of TA. TA-treated *C. albicans* cells were stained with JC-1 dye, and their mitochondrial membrane potentials were detected by flow cytometry. TA-treated *C. albicans* showed a considerably higher ROS value than untreated *C. albicans* (Figure 3A), and 16 μg/mL TA-treated *C. albicans* showed the highest ROS value and the lowest mitochondrial membrane potential (Figure 3B). The mitochondrial membrane potentials of the TA-4 group and TA-8 group were significantly lower than that of the untreated (CK) group (Figure 3B). Meanwhile, these results indicated that ROS burst occurred in TA-treated *C. albicans*, thus causing damage to *C. albicans* cells. 

### 2.5. Transcriptome Sequencing of TA-Treated C. albicans

**Figure 4:**
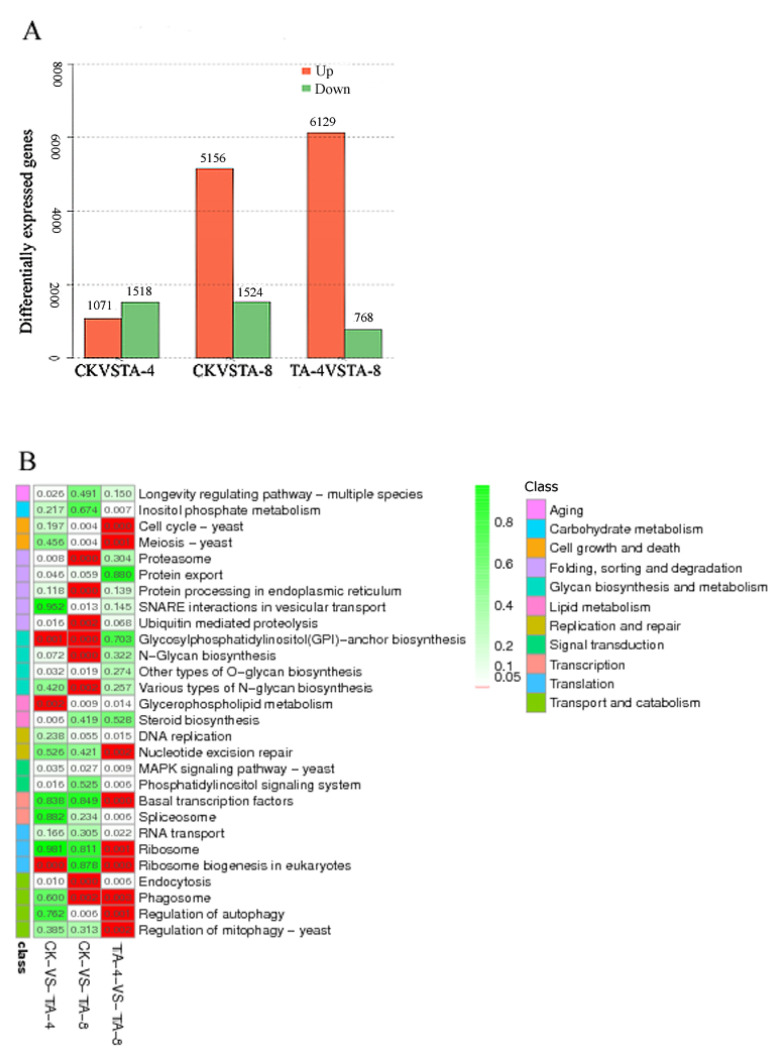
The differentially expressed unigenes and the pathway enrichment in different groups: (**A**) Differentially expressed unigenes; (**B**) KEGG enrichment.

Transcriptome sequencing of TA-treated *C. albicans* was performed to confirm the differentially expressed genes after TA treatment. *C. albicans* was treated with TA-4 and TA-8. The total RNA extracted from the CK group and TA-treated *C. albicans* was sequenced. A total of 16 876 unigenes were annotated with an average length of 913 bp and a GC content of 37.75%. A total of 1518 unigenes were up-regulated and 1071 unigenes were down-regulated in CK relative to in MA-4 (TA-4). Relative to those in MA-8 (TA-8), 5156 unigenes were down-regulated and 1524 unigenes were up-regulated in CK, and 6129 unigenes were down-regulated and 768 unigenes were up-regulated in MA-4 (Figure 4A). The differentially expressed genes between the CK and MA-4 (TA-4) groups were mainly enriched in the pathways of ribosomal biogenesis, glycosylphosphatidylinstiol (GPI)-anchor biosynthesis, glycerolphospholipid metabolism, proteasome, steroid biosynthesis, and endocytosis. The differentially expressed genes between the CK and MA-8 (TA-8) groups were mainly enriched in the pathways of ribosomal biogenesis, endoplasmic reticulum protein, *N*-polysaccharide biosynthesis, GPI-anchored protein biosynthesis, endocytosis, and phagosome-related genes (Figure 4B). These results suggest that TA kills *C. albicans* mainly by destroying the endoplasmic reticulum and ribosome of *C. albicans*, and the proteasomes with up-regulated expression would remove the proteins impaired by TA. Among these genes, 35 genes related to the MAPK signaling pathway were significantly differentially expressed in the MA-8 group relative to in the CK group, indicating the important role of the MAPK pathway in the anticandidal activity of TA.

### 2.6. Proteomic Analysis of TA-Treated C. albicans

In this study, the proteomes of untreated and TA-treated *C. albicans* were analyzed by using iTRAQ technology to navigate the significantly differentially expressed protein after TA treatment. By using the ProteinPilot 5.0 search engine, 14,461 peptides and 2746 proteins with high confidence (confidence level > 95%) and high repeatability (coefficient of variation < 0.5%) were identified on the basis of the transcriptome of TA-treated *C. albicans*. The available proteins had amino acid lengths of 27, 193, 266, 291, 346, 266, 260, 181, 145, 98, and 475 with masses of 0–10, 10–20, 20–30, 30–40, 40–50, 50–60, 60–70, 70–80, 80–90, and >100 kDa, respectively (Figure S2A). The peptide length distribution of the identified proteins showed that nearly 10% of the peptides were 12 amino acids in length (Figure S2B). 

GO and COG analyses were performed on transcriptomic data. GO analysis revealed that most genes were involved in the cellular process, followed by the metabolic process, and then by cellular and catalytic activity processes (Figure S2C). KOG analysis revealed that most genes were involved in post-translational modification and protein turnover, followed by general function, ribosomal structure and biogenesis, and intracelluar trafficking.

**Figure 5:**
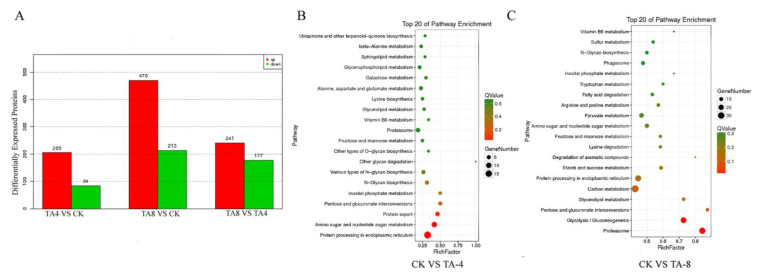
The differential proteins and KEGG pathway enrichment after TA treatment: (**A**) Differentially expressed proteins; (**B**) KEGG pathway enrichment of CK VS TA-4; (**C**) KEGG pathway enrichment of CK VS TA-8.

A total of 205 proteins were up-regulated and 84 proteins were down-regulated in the TA-4 group relative to in the CK group; meanwhile, 470 proteins were up-regulated and 213 proteins were down-regulated in the TA-8 group relative to in the CK group (Figure 5A). The differentially expressed proteins between CK vs. TA-4 (Figure 5B) and CK vs. TA-8 (Figure 5C) were enriched in different pathways. In the CK vs. TA-4 group, protein processing in the endoplasmic reticulum took first place, followed by amino sugar and nucleotide sugar metabolism and the protein export pathway. In the CK vs. TA-8 group, the proteasome ranked first, followed by glycolysis and glycerolipid metabolism, and protein processing in the endoplasmic reticulum ranked sixth, indicating that the cell wall, cell membrane, and the endoplasmic reticulum are the main targets of TA in *C. albicans.*

Differentially expressed proteins with more than 1.2-fold differences and fewer than 0.85-fold (*p* < 0.05) differences between the CK vs. TA-8 and CK vs. TA-4 are listed in Table S1. The transcriptome database showed that the expression levels of 142 proteins increased by more than 1.2-fold and those of 84 proteins decreased by less than 0.85-fold in the TA-4 group relative to those in the CK group. Meanwhile, the expression levels of 405 proteins increased by 1.2-fold and those of 213 proteins decreased by 0.85-fold in the TA-4 group compared with those in the CK group. The most up-regulated proteins in the TA-8 group relative to those in the CK group were Pga31p (anchored component of cell membrane, 4874-fold), supereoxide dismutase 5 (SOD5, 3.504-fold), transglycosylase (intrinsic component of cell membrane, 3.385-fold), inositol-3-phosphate synthase (fungal-type cell wall, 2.979-fold), and glycerol-1-phosphatase (response to stress, 2.66-fold). Meanwhile, the most down-regulated proteins in the TA-8 group were Ucf1p (plasma membrane, 0.232-fold), Blp1p (0.307-fold), AQY1 protein (intrinsic component of cell membrane, 0.339-fold), Asr1p (0.400-fold), and L-methionine-(R)-S-oxide reductase (cysteine and methionine metabolism, 0.488-fold). These results suggest that TA attacks the cell membrane and wall of *C. albicans* and also impairs amino acid metabolism. These effects lead to the up-regulation of some proteins related to the response to a chemical stimulus. The MAPK pathway comparison of different groups indicated that most unigenes in this pathway were up-regulated in TA-treated *C. albicans* compared with those in the CK group (Figure S3).

### 2.7. Validation of Significantly Differentially Expressed Proteins by Western Blot Analysis

**Figure 6:**
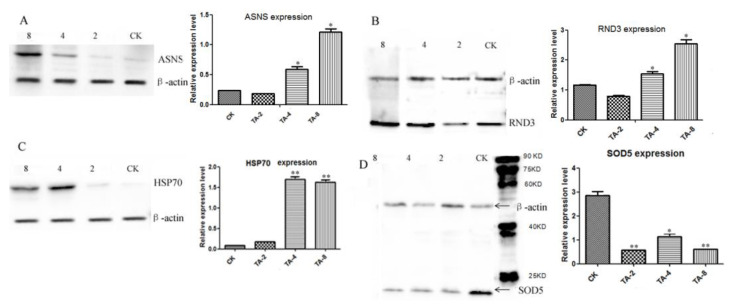
The significantly differentially expressed proteins verified by Western blot after TA treatment: (**A**) ASNS; (**B**) RND3; (**C**) HSP70; (**D**) SOD5. Relative expression level refers to the expression level of target proteins compared to that of β-actin. “*” indicates *p* < 0.05, “**” indicates *p* < 0.01.

The significantly differentially expressed proteins identified by iTRAQ analysis were further validated through Western blot analysis with β-actin as the reference. The proteins of the CK, TA-4, and TA-8 groups were extracted and the expression levels of proteins, including SOD, the Rho-related GTP-binding protein RND3, asparagine synthetase (ASNS), and HSP70, were detected through Western blot analysis. The results indicated that the expression levels of ASNS (Figure 6A), RND3 (Figure 6B), and HSP70 (Figure 6C) were significantly higher in the TA-4 group than in the CK group (*p* < 0.05). Meanwhile, the expression levels of superoxide dismutase (SOD) significantly decreased in TA-treated *C. albicans* (Figure 6D), likely due to the impairment of SOD caused by mitochondrial damage. The expression levels of RND3, ASNS, and HSP70 were considerably higher in the TA-8 group than in the TA-4 and CK groups, indicating the occurrence of defensive responses to toxic stress mediated by these three proteins in *C. albicans.*

## 3. Discussion

*C. albicans* can be a pathogen in humans under certain circumstances, and its drug resistance is increasing. Hence, developing novel anti-*C. albicans* leading compounds is imminent. In this study, TA isolated from *T. spirale* exhibited strong anti-*C. albicans* activity. Comparative transcriptomic and proteomic analyses combined with qRT-PCR and Western blot analyses were performed to investigate the genes and proteins that were significantly differentially expressed after TA treatment. Flow cytometry and ROS detection were also carried out to clarify the cellular mechanism of anti-*C. albicans.* This work is the first to discover that the compound TA showed a prominent inhibitory effect on *C. albicans*. Meanwhile, TA exerted very weak cytotoxicity on the human-derived cell lines, suggesting that TA has great potential for development as an anti-*C. albicans* leading compound. However, we tested the cytotoxicity of TA towards LX-2 derived from liver, which does not reflect any skin or mucous infections, nor any systemic infections. Therefore, the cytotoxicity of TA towards various cell lines including skin or mucous cell lines needs to be further tested to ensure the safety of TA in possible clinical application. The structure of TA shared a unique structure with known anti-candidal drugs, suggesting the possible unique anti-candidal mechanism of TA. 

The Western blot analysis results obtained through transcriptomic and proteomic sequencing showed that in TA treated-*C. albicans*, SOD, GST, RND3, ASNS, and HSP70 were significantly up-regulated, whereas the proteins related to respiratory growth and development and the endoplasmic reticulum were remarkably down-regulated. The above results, in combination with the findings of ROS and michitochondrial membrane potential assays, indicated that the mitochondria, ribosomal proteins, and endoplasmic reticulum were preferentially targeted by TA. Our previous studies showed that 100 μg/mL TA showed no inhibitory effect on various bacteria, including *Escherichia coli*, *Staphylococcus aureus*, *Bacillus subtillis*, and *Pseudomonas aeruginosa*, which was probably due to the absence of organelles, including the endoplasmic reticulum and mitochondria, in bacteria. These findings can further support our hypothesis that the mitochondria and endoplasmic reticulum are the dominant targets of TA in *C. albicans.* Previous studies have shown that nitric oxide (NO) content is closely related to candidacidal activity [20,21,22]. The addition of the NO synthase inhibitor L-monomethylarginine led to the reduced amounts of NO in rat peritoneal neutrophils and weakened antifungal activity, which can be reversed by the oxidation of L-arginine, suggesting that the metabolism of L-arginine is associated with anticandidal activity [20]. The increased amounts of reactive oxygen species (ROS) detected in TA-treated *C. albicans* demonstrated that ROS content is positively related to NO content. Thus, our research findings also suggested that NO, ROS contents and arginine oxidation are the key factors for killing *C. albicans*. Additionally, the gene expression also varies according to the medium, the expression levels of these genes in different medium-cultured *C. albicans* needs to be further investigated in our future study.

Several studies have documented that in eukaryotic organisms, such as *C. albicans*, the mitochondrion is associated with ROS generation [23], apoptosis [24,25], and aging [26]. Meanwhile, ROS produced by the oxidation of Ero1 protein in the endoplasmic reticulum contributes to the toxicity to *C. albicans* via cell wall stress [27]. The scavenging of ROS can attenuate the antifungal effect on *C. albicans.* Miconazole shows anticandidal potency via inducing ROS generation through inhibiting the activity of peroxidases and catalases [9,28]. These findings provide further evidence for our discovery that TA mainly attacks mitochondria to induce ROS production, thereby causing damage to the cell wall and membrane. The expression levels of peroxidases, including SOD and GST, were significantly up-regulated to alleviate ROS-induced cell wall stress in *C. albicans* under TA treatment. 

RND3 functions in the organization of actin filaments and the regulation of actin cytoskeleton organization, as well as cell migration and cell shape formation [29,30]. The increase in the expression level of RND3 in *C. albicans* after TA treatment suggested the occurrence of cytoskeleton organization and cell reshaping to maintain the integrity of *C. albicans* to resist TA toxicity. HSP70 is involved in a wide variety of cellular processes, including the protection of the proteome from stress [31]. This situation indicated that the up-regulation of HSP70 in TA-treated *C. albicans* resulted in the protection of the proteome from stress, thereby promoting the correct folding of proteins and reducing degradation in TA-treated *C. albicans* through the process of ATP binding. ASNS catalyzes the synthesis of asparagine and glutamate in an ATP-dependent manner. The GO functions of ASNS include a response to toxic substances, suggesting that the intracellular up-regulation of ASNS would mitigate the toxicity of TA to *C. albicans.* In wheat seedlings, ASNS has been demonstrated to be significantly up-regulated by salinity, osmotic stress, and exogenous abscisic acid [32]. Our findings also indicate the important role of ASNS in the defensive response of *C. albicans* to TA treatment. 

Several studies have been conducted to exploit novel compounds with anticandidal activity and to elucidate their mechanisms. Treatment with high concentrations of perillyl alcohol led to the diminished virulence, cell cycle arrest, and mitochondrial dysfunction of *C. albicans* [33]. Moreover, caspofungin and ferulic acid exert synergistic apoptotic and anticandidal effects on *C. albicans* [34]. However, previous studies have failed to discover new compounds with very potent anticandidal activity [35,36,37,38,39]. The present study is the first to report that TA demonstrates highly potent anticandidal activity and sheds light on the development of a novel leading compound to kill *C. albicans.* The discovery of 49 significantly differentially expressed genes, including SOD and iron-uptake-related genes, in ciclopirox olamine-treated *C. albicans* demonstrated the function of ciclopirox olamine as an iron chelator and ROS generation inducer [27]. Moreover, macrolide antibiotics, including erythromycin, clarithromycin, azithromycin, and spiramycin, have been widely used in clinical treatment due to their low side effects [40]. Macrolide antibiotics can inhibit protein synthesis and elongation by binding to 50 major subunits of bacterial ribosomes. The ability of TA to bind preferentially to the mitochondrial ribosomal units of *C. albicans* may provide clues for the weak inhibitory effect of TA to bacteria, including *E. coli*, *Bacillus subtilis*, and *Staphylococcus aureus*.

**Figure 7:**
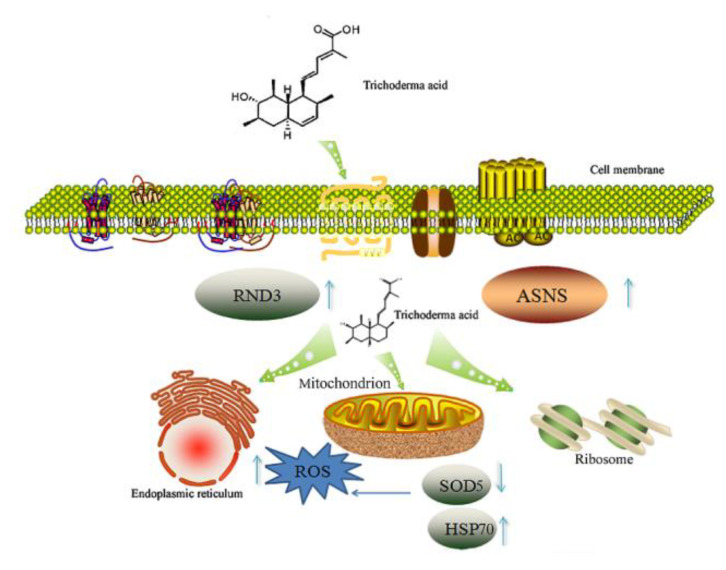
The proposed action mechanism of TA towards *C. albicans*.

The anti-*C. albicans* mechanism of TA is proposed as follows: First, TA treatment impairs the cell membrane and cell wall structure of *C. albicans* while down-regulating the expression of SOD, leading to ROS accumulation. The high ROS concentration leads to DNA damage and cell skeleton destruction, resulting in the up-regulation of proteins related to cell skeleton integrity, such as RND3 and ASNS. The cell membrane is then attacked by the elevated concentration of TA. The organelles including the endoplasmic reticulum and mitochondria in *C. albicans* are attacked subsequently, and peroxidases, including GST, are up-regulated to protect *C. albicans* cells from the hazard of TA via ROS. Meanwhile, the expression of some proteasomes are up-regulated to remove the proteins degraded by TA (Figure 7). Proteins in the MAPK signaling pathway, including Swi4.6, which is related to cell wall remodeling; Rho1, which is related to hypotonic shock; and Glo1, which is related to osmolyte synthesis, are also up-regulated to mitigate the adverse effects caused by TA (Figure S3).

The network of 79 significantly differentially expressed proteins between the CK and TA-8 groups with changes of more than 1.2-fold and less than 0.85-fold was constructed using the software STRING 11.5 (Figure S4). The differentially expressed proteins were divided into three clusters. In the red cluster, CaO19.2788, which refers to adaptins that are assumed to interact with membrane proteins and CEK2 proteins; CDC24, which refers to the MAPK pathway protein Rho protein; and YCF1, which refers to the ATP-binding cassette glutathione S-conjugate transporter, showed the highest number of nodes with other proteins. These results suggested the important role of the MAPK pathway and GST in the defensive response of *C. albicans* to TA treatment. In the green cluster, RPL27 A, which encodes the 60S ribosomal protein L27, and RPL3, which encodes the 60S ribosomal protein L3, had the highest number of interactions with other proteins. These results indicated that ribosomal proteins are the potential target of TA. In the blue cluster, E2 ubiquitin-conjugating protein UBC4, proteasome regulatory particle lid subunit RNP7, and ubiquitin-conjugating enzyme family CaO1954.11 shared the most nodes, suggesting that they are potential targets of TA. The thiopeptide thiostrepton shows a strong inhibitory effect on the pathogenic bacterium *Mycobacterium marinum* by binding to the cleft between the L11 protein and 23S rRNA of the 50S large ribosomal subunit, thus hindering the translation and the subsequent protein synthesis of *M. marinum* [41,42]. The present study hints that the 60S ribosomal protein L3 and L27 are the potential targets of TA in *C. albicans.*

## 4. Materials and Methods

### 4.1. The Strains, Cells and Compounds

The strain *C. albicans* (ATCC10231) was purchased from ATCC. *Trichoderma spiralis* A17, an endophytic fungus derived from *Aquilaria sinensis*, originated from Nantun village, Xinyi, Guangdong Province. The ITS sequence was obtained and deposited in NCBI (Genbank No. EU781674.1). which showed 100% identity with the ITS sequence from *Trichoderma spirale* isolate y-038, suggesting the A17 strain as Trichoderma spirale. The morphological feature of A17 strain was characterized as follows: the hyphae grew densely and were white and cotton-like at the early stage. The hyphae had a septum, extending upward into an erect conidia, which divided into two opposite lateral branches and finally formed a stem. 

The cell line LX-2 (Human hepatic stellate cells) were purchased from the cell bank of the Chinese Academy of Sciences. The crude extract of *T. spiralis* A17 was obtained by the addition of an equal volume of ethyl acetate into the fermentation liquid of *T. spiralis* A17 and then concentrated via rotary evaporation, which was repeated three times, and then the compound TA with the purity higher than 98% was isolated via column chromatography from the crude extract of *T. spiralis* A17 [18]. 

### 4.2. Anti-C. albicans Activity Assay

The inhibitory effect of TA towards *C. albicans* was assayed using the disc diffusion method [43]. Briefly, *C. albicans* cells were diluted to a concentration of 10^7^ cells/mL, 200 μL *C. albicans* suspensions were spread on potato dextrose agar (PDA) plate evenly, and the inhibition zone was measured after 48 h cultivation. Then, 5 μL solution of TA or *T. spiralis* A17 crude extract was dropped onto a 6 mm diameter filter paper disk which was put on the surface of a PDA plate. Additionally, the concentrations of TA and *T. spiralis* A17 crude extract were 20 μg/mL and 50 μg/mL, respectively. The growth curves of *C. albicans* treated with TA were determined using a microplate reader (Biotek, Winooski, VT, USA). 

### 4.3. Cytotoxicity Assay

The in vitro cytotoxicity of TA towards human-derived cell line LX-2 (Human hepatic stellate cells) was assayed using the SRB (Sulforhodamine B) method [44]. Briefly, the tested cell line LX-2 was fetched from a −80 °C fridge before completely thawing it. Then, it was passaged for three generations when the cells’ coverage was 80% in DMEM medium containing penicillin–streptomycin solution (the concentration of penicillin was 100 U/mL and the concentration of streptomycin was 0.1 mg/mL), 10% fetal bovine serum and streptomycin. After the trypsin treatment and the viability detection by trypan blue (viability > 95%), the cells were adjusted to 3 × 10^6^ cells/mL by fresh DMEM medium, which was then seeded into 96-well plates and incubated at the condition of 37 °C and 5% CO_2_ for 24 h.

Then, different concentrations of TA with the dosage of 1–128 μM were added and co-incubated at the condition of 37 °C and 5% CO_2_ for another 72 h. After being immobilized with 50 μL 50% cold TCA (trichloroacetic acid), stained with 100 μL SRB with the concentration of 4 mg/mL for 30 min, and washed with 1% acetic acid 5 times, the cells were dissolved in 200 μL 10 mM Tris base solution and the OD at 570 nm was recorded using a THERMOmax microplate reader (Molecular Devices, Sunnyvale, CA, USA) to assay the cytotoxicity of TA. Additionally, adriamycin was used as a positive control. All data were acquired in triplicate, and the IC_50_ values were calculated using the SigmaPlot 10.0 software (Systat Software Inc., San Jose, CA, USA) with the use of a non-linear curve-fitting method. 

### 4.4. The Flow Cytometry, Laser Confocal Microscopy Analysis and ROS Detection

*C. albicans* was cultivated in yeast extract peptone dextrose (YPD) medium for 16 h, then adjusted to 1 × 10^6^ cells/mL. TA with concentrations of 4 μg/mL, 16 μg/mL, 32 μg/mL, and 64 μg/mL were added to the YPD medium, then co-cultured with *C. albicans* for 24 h. The cells of *C. albicans* were collected after centrifugation at 12000 rpm, and then washed twice with PBS. After the addition of 500 μL 10 μg/mL DCFH-DA (Thermo Fisher Scientific Inc., San Francisco, CA, USA), the *C. albicans* cells were placed at room temperature in the dark to stain for 30 min, and then the cells were observed via laser confocal microscopy with a reading parameter of Ex/Em = 488/525 nm and a magnification fold of ×400 (Axio-Imager_LSM-700, Zeiss, Oberkochen, Germany). Additionally, the reactive species oxygen (ROS) value of *C. albicans* was determined using a FACS Calibur flow cytometer and Cell Quest software 3.3 (BD Biosciences, San Jose, CA, USA). The *C. albicans* cells were collected, and 500 μL JC-1 (Ex/Em = 510/527 nm) with a concentration of 10 μg/mL (Thermo Fisher Scientific Inc., San Francisco, CA, USA) was added to measure the mitochondrial membrane potential of *C. albicans* treated with TA [45]. The collected cells were stained with dyes of SYTO-9 and PI, and the *C. albicans* cells were placed at room temperature in the dark to stain for 30 min, which was then detected using a FACS Calibur flow cytometer and Cell Quest software (BD Biosciences, San Jose, CA, USA). The MIC_50_ and MIC_80_ values of TA were measured using flow cytometry, and nystatin was employed as a positive control. 

### 4.5. The Transcriptomic Analysis of TA-Treated C. albicans 

The *C. albicans* strain was cultured in a YPD medium for 12 h, and TA was added with concentrations of 4 μg/mL (TA-4) and 8 μg/mL (TA-8). After being treated with TA for 12 h, the cells of TA-treated *C. albicans* were collected, and RNA was extracted with an RNA extraction kit (Umagen, Guangzhou, China). The integrity of RNA was detected using HPLC (Aglient 2100, Santa Clara, CA, USA). The two cDNA strands were synthesized using a cDNA synthesis kit (New England Biolabs, Hitchin, UK). Then, the poly-A was added using NEB Next End prep enzyme and blunt TA ligase and NEB Next Adaptor to prepare the cDNA library. The cDNA library was constructed using the Illumina sequencing platform (Illumina HiSeq™ 2000) and single-end paired-end (PE) technology. PE reads with a length of 90 bp were obtained via the Illumina GA Pipeline software (version 1.6). All the transcriptomes of different samples were assembled. The low-quality sequences were removed by a Perl. The contigs and transcripts were assembled from high-quality sequences, as well as annotated via the NCBI database combined with the CLC NGS Cell software. The resulting unigenes were determined from COG and GO terms and then further analyzed via the KEGG database. All the transcriptomic sequencing of different groups including CK, TA-4 and TA-8 were conducted in biological triplicate. The differentially expressed genes were analyzed using edgeR software 3.16 (https://www.bioconductor.org/packages/release/bioc/html/edgeR.html, accessed on 10 May 2020) according to the reads per kilobase of exon (RPKM) value [46]. Additionally, all the transcriptomic raw data was deposited in the NCBI database with accession No. SRR14297815. 

### 4.6. The Differentially Expressed Unigenes in TA-Treated C. albicans

Differentially expressed genes (DEGs) were annotated using Blast2GO software. We used WEGO software to acquire the GO functional classifications for all DEGs. The number of uniquely mapped RPKM was used to indicate the gene expression level. The differential expression was featured by the fold changes among unigene expression levels. The *p*-value of <0.05 was defined as the significant differential expression.

### 4.7. The Proteomic Assay of TA-Treated C. albicans

The samples of CK, TA-4, and TA-8 were collected, the cells of TA-treated *C. albicans* were then vortexed with extraction buffer including 0.1 mg PVPP, 2% β-mercaptoethanol, 1 mM preparations of phenylmethylsulfonyl fluoride (PMSF) in 0.5 M Tris-HCl buffer supplemented with 0.7 M sucrose, 0.05 M EDTA, and 0.1 M KCl. Precipitation of all the proteins were obtained by the addition of 5 vol of cold methanol containing 0.1 M ammonium acetate at low temperature overnight. The proteins were dissolved with methanol twice and then centrifuged at 12,000× *g* for 20 min at 4 °C. The proteins were resuspended in 50 mM Tris-HCl buffer supplemented with NaCl 150 mM, SDS 1%, Triton X-100 1%, and 1 mM PMSF pH 8.8, and then centrifuged before acetone and 10% TCA was added. Finally, the pellets were dried and resuspended in the above 50 mM Tris-HCl buffer.

The proteins were reduced using a tris (2-carboxyethyl) phosphine (TCEP) reducing agent and incubated at 60 °C for 1 h (iTRAQ 8-plexkits, AB Sciex, Foster City, CA, USA). We then added cysteine-blocking reagent and transferred the proteins to 10-kDa cut-off ultra-filtration, followed by the addition of 8 M urea and 0.25 M tetraethylammonium bromide (TEAB) (pH 8.5). Then, 2% (*w*/*w*) trypsin was supplemented and incubated at 37 °C for overnight, and then trypsin was added (1%, *w*/*w*) at 37 °C for 4 h. The digested peptides were then collected after centrifugation. iTRAQ labeling was performed according to the manufacturer’s instructions (Applied Biosystems, Waltham, MA, USA). The labeling of CK, TA-4, and TA-8 samples was conducted using different molecular weight iTRAQ tags, and the samples were then incubated at room temperature for 2 h, before being pooled and vacuum-dried. The LC-MS/MS analysis was implemented using a Q Exactive MS. All the proteomic sequencing processes of different groups including CK, TA-4, TA-8 were conducted in biological duplicate. The protein was defined according to the blast results with the transcriptomic data and UniProt database. All the proteomic raw data were deposited in the iProX database (https://www.iprox.org/page/MSV022.html, accessed on 10 May 2022) with accession No. IPX0003029000. The mass spectrometry proteomics data were deposited in the ProteomeXchange Consortium via the iProX partner repository with the dataset identifier PXD025834. 

### 4.8. Protein Identification and Data Analysis

We converted the raw LC-MS/MS detection files into the MASCOT generic format (.mgf) files, and we deposited the mass spectrometry proteomics data in the database of ProteomeXchange. We used ProteinPilot 5.0 software (AB Sciex, Foster City, CA, USA) to execute the proteome analysis and protein quantitation analysis. Additionally, we also this Protein Pilot 5.0 software to search databases and screen reasonable ratios (Unused1.3) [47]. The differential proteins with credibility were screened based on the transcriptome-annotated proteins of various TA-treated *C. albicans*. Proteins with different abundances were classified based on the GO and KEGG database to predict biological processes, molecular function and significant pathways involved in cell structure caused by ROS and the defensive response of *C. albicans* to TA treatment.

### 4.9. The Western Blot Analysis

The most significant differentially expressed proteins identified from the proteomic analysis were verified via Western blot analysis. The total proteins for proteomic analysis were detected via SDS-PAGE and transferred onto an NC membrane. The corresponding primary antibody from mice (Cambridge, UK) was added with a dilution of 1:5000. Five-percent non-fat milk was added to block the non-specific protein, and then secondary antibody (goat anti-mouse IgG) (Fermentas, Hanover, MD, USA) was added with a dilution of 1:8000 after washing. β-actin was used as a reference. The target bands were visualized using an ECL (Electro Chemical Luminescence) kit (Fermentas, Hanover, MD, USA) following the manufacturer’s instructions.

### 4.10. Statistical Analysis

A probability value of *p* < 0.05 in all the tests was judged as significant. Statistical analysis was conducted using the GraphPad Prism version 5.0 software (Graph Pad Software Inc. 1998, San Diego, CA, USA). A t-test was used in our statistical analysis. The expression levels of different proteins in Western blot were judged using Image J (Version 1.8.0) (National Institutes of Health, Bethesda, MD, USA). All experiments were conducted in triplicate.

## 5. Conclusions

In summary, TA isolated from *T. spiralis* exhibited a strong and specific inhibitory effect towards *C. albicans*. Thus, TA was recognized as a promising leading compound for the development of anti-*C. albicans* drugs. SEM observation combined with comparative transcriptomic and proteomic analyses suggested that in *C. albicans*, TA attacks the cell membrane and wall, as well as the endoplasmic reticulum and the ribosomal subunits in the mitochondria, possibly by targeting respiratory growth induced protein 1, homeobox leucine-zipper protein, and butanediol dehydrogenase, subsequently repressing SOD5 and causing ROS accumulation. These effects consequently lead to the destruction of the cell skeleton. The expression levels of GST, RND3, ASNS, and HSP70 were significantly up-regulated as a response to apoptosis and toxic stimuli. The investigation on the toxic mechanism of TA in *C. albicans* could lay a foundation for the development of TA as an anti-*C. albicans* leading compound that would thereby alleviate the hazard of *C. albicans* to human health.

## Data Availability

The data presented in this study are available on request from the corresponding author. The data are not publicly available due to privacy. Additionally, all the transcriptomic raw data were deposited in the NCBI database with accession No. SRR14297815. All the proteomic raw data were deposited in iProX database (https://www.iprox.org/page/MSV022.html, accessed on 10 May 2022) with accession No. IPX0003029000. The mass spectrometry proteomics data have been deposited in the ProteomeXchange Consortium via the iProX partner repository with the dataset identifier PXD025834.

## Supplementary Materials

The following supporting information can be downloaded at: https://www.mdpi.com/article/10.3390/ijms24065445/s1.
