# Supplementary material for: Potential Anti-Candida albicans Mechanism of Trichoderma Acid from Trichoderma spirale
Source: Int J Mol Sci. 2023 Mar 13;24(6):5445. doi: 10.3390/ijms24065445 (PMC10049406; doi:10.3390/ijms24065445)
Supplement: Supplementary file 1 [file ijms-24-05445-s001.zip › Supplementary materials.pdf]

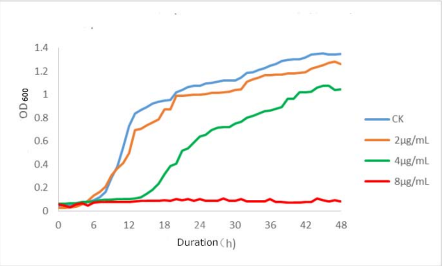

Figure S1 The inhibition of the growth of *C. albicans* by TA

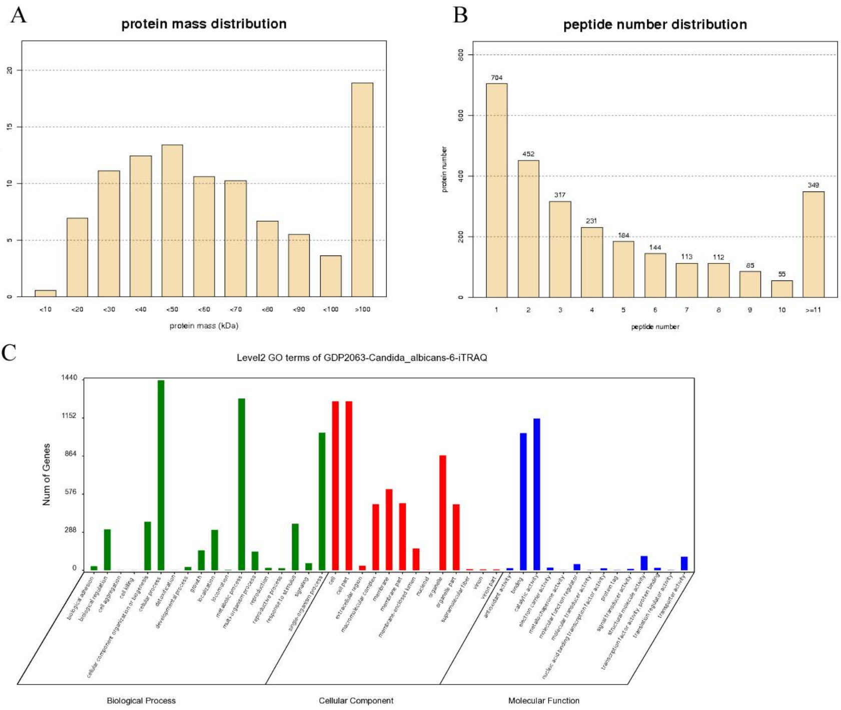

Fig. S2 The peptide distribution and GO functional calssification of proteomic analysis

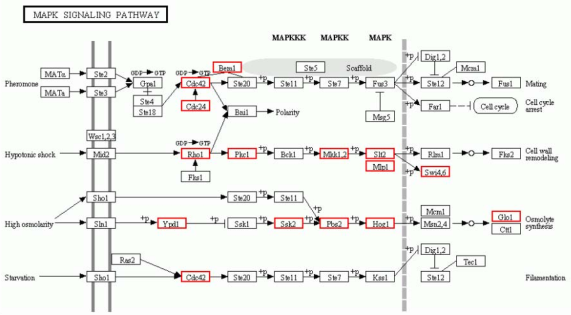

Fig. S3 The KEGG map of MAPK pathway in TA treated *C. albicans*

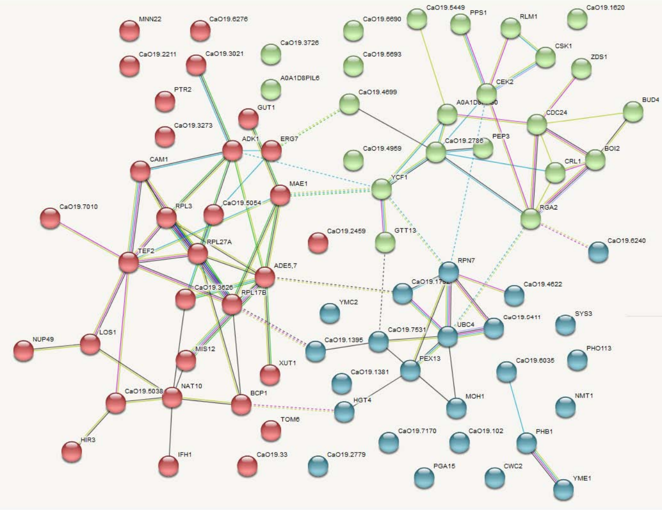

Fig. S4 The networking of differential expressed proteins in TA treated *C. albicans*
